# Availability and Temporal Heterogeneity of Water Supply Affect the Vertical Distribution and Mortality of a Belowground Herbivore and Consequently Plant Growth

**DOI:** 10.1371/journal.pone.0100437

**Published:** 2014-06-17

**Authors:** Tomonori Tsunoda, Naoki Kachi, Jun-Ichirou Suzuki

**Affiliations:** Department of Biological Sciences, Tokyo Metropolitan University, Hachioji, Tokyo, Japan; University of Tartu, Estonia

## Abstract

We examined how the volume and temporal heterogeneity of water supply changed the vertical distribution and mortality of a belowground herbivore, and consequently affected plant biomass. *Plantago lanceolata* (Plantaginaceae) seedlings were grown at one per pot under different combinations of water volume (large or small volume) and heterogeneity (homogeneous water conditions, watered every day; heterogeneous conditions, watered every 4 days) in the presence or absence of a larva of the belowground herbivorous insect, *Anomala cuprea* (Coleoptera: Scarabaeidae). The larva was confined in different vertical distributions to top feeding zone (top treatment), middle feeding zone (middle treatment), or bottom feeding zone (bottom treatment); alternatively no larva was introduced (control treatment) or larval movement was not confined (free treatment). Three-way interaction between water volume, heterogeneity, and the herbivore significantly affected plant biomass. With a large water volume, plant biomass was lower in free treatment than in control treatment regardless of heterogeneity. Plant biomass in free treatment was as low as in top treatment. With a small water volume and in free treatment, plant biomass was low (similar to that under top treatment) under homogeneous water conditions but high under heterogeneous ones (similar to that under middle or bottom treatment). Therefore, there was little effect of belowground herbivory on plant growth under heterogeneous water conditions. In other watering regimes, herbivores would be distributed in the shallow soil and reduced root biomass. Herbivore mortality was high with homogeneous application of a large volume or heterogeneous application of a small water volume. Under the large water volume, plant biomass was high in pots in which the herbivore had died. Thus, the combinations of water volume and heterogeneity affected plant growth via the change of a belowground herbivore.

## Introduction

Water availability and its temporal variability (hereafter, ‘water heterogeneity’) affect plant biomass growth [1], [2]. Plant responses to water frequency vary depending on nutrient availability and soil water content [3]–[6]. Soil water status also affects soil biota [7], and interacts with soil biota to affect plant growth. Empirical studies are necessary to clarify the interactive effects of water heterogeneity on soil biota and hence on plant growth because of the important influence of soil biota on plant growth and community dynamics [7], [8].

In light of the presence of herbivorous insects in the soil, it is important that we elucidate the effects of water availability and heterogeneity on plants [9], because these insects influence the abundance, species diversity, and succession of plants [10]–[14]. Erb and Lu [15] pointed out that heterogeneity of soil abiotic factors such as moisture and nutrient availability alters the effects of belowground herbivores on plants. The interactions between water availability, heterogeneity, and belowground herbivory are likely to play crucial roles in plant growth.

Water availability and frequency affect the vertical distribution of belowground herbivores, and thus plant growth, because soil insects move vertically in response to changes in soil water status [16], [17]. Grubs (Coleoptera: Scarabaeidae) are distributed deep in the soil in response to drought and shallow in the soil in response to irrigation [18]. The carrot-fly larva *Psila rosae* feeds on roots 15 cm below the ground in semi-dry soil, whereas in moist soil it feeds 1 cm from the soil surface [19]. Wireworms (Coleoptera: Elateridae) are distributed deep in the soil in summer but in shallow soil after heavy rain [20]. These changes in the vertical distribution of belowground herbivores affect plant mortality and growth [21]. Therefore, the amount and heterogeneity of water supply that determine soil moisture levels are likely to affect the vertical distribution of belowground herbivores and thus plant growth.

Soil moisture is one of the most important factors affecting the survival and abundance of belowground herbivorous insects [16], [17], [22]. Soil dryness increases mortality of belowground herbivores [23]–[26], whereas in moist soil mortality either does not change [24], [27], [28] or increases [23], [29]. These findings suggest that the mortality of belowground herbivores in response to extreme water events has the potential to affect plant growth in various ways.

We conducted a growth experiment to test the hypothesis that changes in the amount and heterogeneity of water supply alter the vertical distribution and mortality of belowground herbivores and thus affect plant growth. Under heterogeneous supply of small amount water, belowground herbivores will become distributed deep in the soil to avoid the dry surface soil and feed on the fine root tips, which will hardly affect plant growth. In contrast, under homogeneous supply of large volume of water, herbivores will become distributed shallow in the soil and detach root connection by grazing, which has impact on plant growth. Soil water status resulting from changes in water supply amount and heterogeneity will also determine the fate of belowground herbivores. Unless belowground herbivores are present, plant growth will be no longer restricted by belowground herbivory.

## Materials and Methods

### Study species

One seedling of *Plantago lanceolata* L. (Plantaginaceae) was grown in each pot, to which we added a third-instar larva, or grub, of the belowground herbivorous insect *Anomala cuprea* Hope (Coleoptera: Scarabaeidae). The short-lived perennial forb, *P. lanceolata*, is cosmopolitan and has a rosette growth form. Seeds of *P. lanceolata* were collected from a population of more than 30 plants on a floodplain of the Tama River in Tokyo (35°38′N, 139°23′E). *P. lanceolata* is not endangered or protected species, and no specific permissions were required for this location to collect seeds of *P. lanceolata*. Larvae of *A. cuprea* feed on various herbaceous species [30], [31], including *P. lanceolata* (T.Tsunoda, personal observation). Grubs were grown from eggs laid in humus by adult *A. cuprea* collected from a floodplain of the Tama River (35°38′N, 139°23′E) in June and July 2012. *A. cuprea* is not endangered or protected species, and no specific permissions were required for this location to collect insects.

### Experimental design

The growth experiment was conducted from September to October 2012 in a plastic film greenhouse under natural sunlight in the experimental garden of Tokyo Metropolitan University (Hachioji, Tokyo; 35°37′N, 139°23′E). The mean annual precipitation in Hachioji has been 1602.3 mm year^–1^ over the past 30 years [32]. Seeds of *P. lanceolata* were sown in a tray of peat moss in a growth chamber (Koitotron PC-02, Koito Industries, Ltd., Kanagawa, Japan) at 25°C. Seven days after sowing, one seedling with cotyledons was transplanted into each plastic pot (20 × 20 × 18 cm deep). Each pot was filled with 4.8 L of a mix of granular red clay and black soil (ratio 1:1 v/v) with 16 g of slow-release fertilizer [Magamp K, 6:40:6:15 (N-P-K-Mg), Hyponex Japan, Osaka, Japan].

The experiment had a three-way factorial randomized block design with nine replications. The factors were water volume with two levels (large, 200 mL of water per day, and small, 100 mL of water per day), water supply frequency with two levels (homogeneous, daily watering, and heterogeneous, watering every 4 days), and belowground herbivore. The certain amount of water for any single watering was determined not to exceed the capacity of the pot. The total volume of water received over the period was the same between the homogeneous and heterogeneous supplies for each water volume regime.

There were five types of belowground herbivore treatment: in the top treatment, one grub was placed in the top zone; in the middle treatment, it was placed in the middle zone; and in the bottom treatment, it was placed in the bottom zone. In the control treatment, no grub was added to the pot. In the free treatment, no screens were added to restrict the grub movement. Two 20 × 20 cm stainless-steel wire-mesh screens (0.8-mm wire diameter, 5.5-mm mesh) were inserted into the pot soil to divide the soil evenly into three zones in all treatments except the free treatment. In the top, middle, and bottom treatments, one grub was introduced to the relevant zone through a hole (diameter 12 mm) on the side wall of the pot. The hole was closed with plastic film after the grub had been added. In the free treatment in which the grub was able to move freely around the pot, four screens of 20 × 10-cm stainless-steel wire-mesh were inserted, one along each wall of the pot, which assumed perfect mobility of the grub without any other differences from other three treatments. Because the insertion of the wire-mesh did not affect the plant growth in our previous experiment [33], no treatment without wire-mesh was included in this experiment.

For the first week after transplantation of the seedlings, 150 mL water was supplied to all pots every day. The treatments that combined different water volumes with different watering frequencies began at the start of the second week after the transplant. On day 20 after the beginning of the watering treatment, a grub was added to the relevant zone of the pot. In the free treatment, the grub was placed on the soil surface near the centre of the pot and left to burrow underground.

A plant survival was recorded every day after addition of the grub. If the roots became completely detached from the shoot and the leaves wilted, the plant was considered to die. When a plant died, we recorded survival of the grub.

On day 28 after addition of the grub, the plants were harvested and divided into shoots and roots. The shoots and roots were dried at 72°C for 3 days and weighed. At harvest, grub mortalities were also recorded.

### Soil moisture measurement

Soil moisture (water content by volume) was measured with a soil moisture probe (ECH_2_O, Decagon Devices, Inc., Pullman, Washington, USA) in an additional four replications without grubs of each watering treatment combination and soil zone during the experimental period. Measurements were taken every day, before the watering. Relative soil moisture content was calculated as the difference between the measured value and the minimum value during the experimental period, divided by the range between the maximum and minimum values during the experimental period [34]. The 4-day moving variance of the relative soil moisture content for the current day and the next 3 days was calculated to quantify the variability in water availability in each 4-day watering cycle. The coefficient of variation (CV) and temporal mean of the relative soil moisture content during each watering treatment were calculated. The CV was used as an index of temporal variability in soil moisture content during each watering treatment [34].

### Data analysis

Relative soil moisture content and temporal variability in soil moisture were analyzed by using generalised linear mixed models (GLMMs) under the assumption of a Gaussian error distribution. In this model, the response variable was the relative soil moisture content or the CV; the explanatory variables were water volume, watering frequency, soil zone, and their interactions: the random factors were measuring date and block, with block as nested random effect within date.

Mortalities of plants and grubs were analyzed by using GLMMs with a binomial error distribution and logit-link function. In these models, the response variable was grub or plant mortality; the explanatory variables were water volume, watering frequency, belowground herbivore, and their interactions. The random factor was block.

Plant biomass as the sum of shoot and root biomass of each plant, was analyzed with a GLMM assuming a Gaussian error distribution. In this model, the response variable was plant biomass; the explanatory variables were water volume, watering frequency, belowground herbivore, and their interactions. The random factor was block. For each water volume, plant biomass was analyzed with the model lacking water volume as an explanatory variable.

In the analyses for continuous variables, if the assumption of homogeneity of variance was satisfied with Bartlett's test, then identity-link function was applied. If not, then log-link function was applied. Data from pots in which belowground herbivores died were treated as missing values. Number of samples in the biomass analyses was presented in Table 1.

**Table 1:** Number of replicates, dead plants, and dead belowground herbivores, and the total sample size in biomass analyses of combinations of experimental treatments.

Water volume	Water heterogeneity	Belowground herbivore	Number of replicates	Number of dead plant	Number of dead belowground herbivore	Sample size in biomass analyses
Small	Homogeneous	Control	9	0	NA†	9
		Bottom	9	0	3	6
		Middle	9	0	0	9
		Top	9	2	1	6
		Free	9	0	1	8
	Heterogeneous	Control	9	0	NA†	9
		Bottom	9	0	4	5
		Middle	9	0	2	7
		Top	9	0	1	8
		Free	9	0	4	5
Large	Homogeneous	Control	9	0	NA†	9
		Bottom	9	0	2	7
		Middle	9	0	6	3
		Top	9	0	4	5
		Free	9	2	3	4
	Heterogeneous	Control	9	0	NA†	9
		Bottom	9	0	2	7
		Middle	9	0	2	7
		Top	9	1	2	6
		Free	9	1	3	5

† NA means not available due to the control treatment.

Because several grubs were found dead at harvest, plant biomass was analyzed with generalised linear models (GLMs) for each water volume to evaluate changes in plant biomass due to herbivore mortality. In these models, the response variable was plant biomass; the explanatory variables were watering frequency, belowground herbivore, the survival of the belowground herbivore, and their interactions.

All analyses were performed with the statistical software R version 2.15.1 [35]. The lme4 package was used to calculate GLMMs by using maximum likelihood estimation. To determine the effects of the fixed factors (i.e., to calculate the in *P*-values), we used a likelihood ratio test to compare models with and without the variable of interest using a chi-squared test statistic [36]. The data were analyzed by GLMM framework because our data contain binary and continuous variables [37].

## Results

### Soil moisture

**Figure 1:**
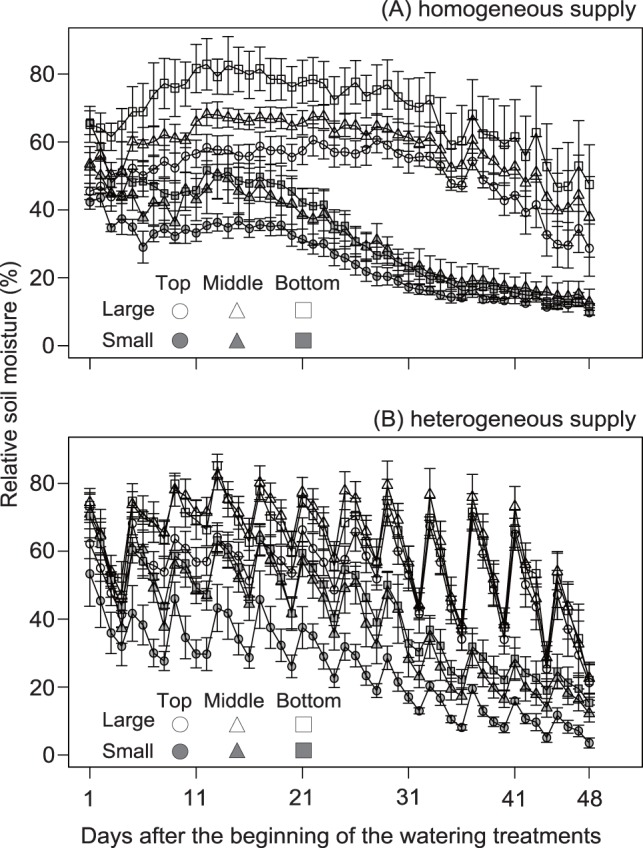
Mean relative soil moisture (± SE). (A) heterogeneous supply treatments; (B) homogeneous supply treatments; Top, middle, bottom: top, middle, and bottom zone treatments, respectively. Large, large water volume supply; Small, small volume supply. The soil moisture content was larger with a large water volume than a small volume. The soil moisture content in the bottom zone was the largest, and that in the top zone the smallest. The temporal variability in soil moisture content under heterogeneous water-supply conditions was larger than that under homogeneous conditions.

The mean relative soil moisture content was larger under a large water volume than a small volume (Figure 1, Table 2). The mean relative soil moisture content in the bottom zone was the largest, and that in the top zone was the smallest (Figure 1; Table 2).

**Table 2:** Effects of water volume (V), water heterogeneity (H), and soil profile (P) on mean relative soil moisture content.

Source	*χ* ^2^	df	P
V	38.788	1	<0.001
H	0.108	1	0.742
P	6.048	2	0.049
V × H	0.822	1	0.365
V × P	0.660	2	0.719
H × P	0.551	2	0.759
V × H × P	0.599	2	0.741

The temporal variability in relative soil moisture content under heterogeneous water-supply conditions was larger than that under homogeneous conditions (Figure 1). The mean CV (%) of the relative soil moisture content was larger under heterogeneous water-supply conditions than under homogeneous conditions (Table 3).

**Table 3:** Effects of water volume (V), water heterogeneity (H), and soil profile (P) on mean CV of the relative soil moisture.

Source	*χ* ^2^	df	P
V	0.333	1	0.564
H	6.425	1	0.011
P	0.252	2	0.882
V × H	0.156	1	0.693
V × P	0.230	2	0.891
H × P	0.081	2	0.960
V × H × P	0.040	2	0.981

### Plant mortality

Plant mortality changed significantly with differences in the feeding zones of the belowground herbivore (Table 4). No plants died in the middle and bottom treatments, but three died in the top treatment and three in the free treatment. No plants died when a small volume of water was heterogeneously applied (Table 1). Roots of the survived plants reached the bottom zone at the harvest.

**Table 4:** Effects of water volume (V), water heterogeneity (H), and belowground herbivore (B) on plant mortality.

Source	*χ* ^2^	df	P
V	0.753	1	0.386
H	0.763	1	0.382
B	8.621	3	0.035
V × H	6.234	3	0.101
V × B	2.156	1	0.142
H × B	0.000	3	1.000
V × H × B	0.000	3	1.000

### Plant biomass

**Figure 2:**
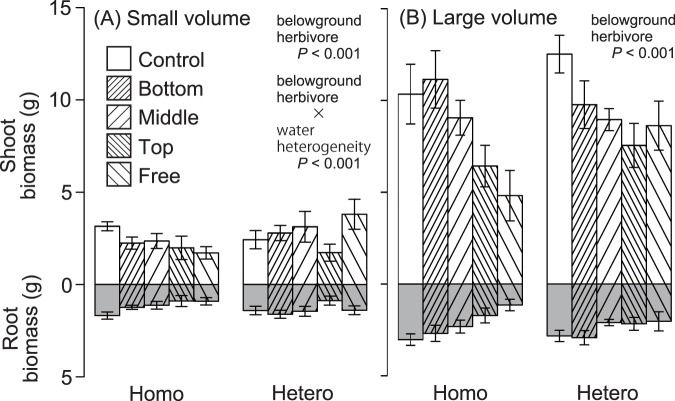
Mean of the shoot and root biomass (± SE). (A) Small water volume. (B) Large water volume. Control, no herbivore present; Bottom, Middle, Top; herbivores placed in the bottom, middle, and top zones, respectively, of the pot; Free, herbivore present and free to move in all zones of the pot. Homo, homogeneous water supply; Hetero, heterogeneous water supply. Biomass in the top treatment was the smallest between the middle, bottom, and control treatments in every watering treatment. Biomass in the free treatment was similar to that in the top treatment in every watering treatment, except for the heterogeneously-applied treatment with small volume of water.

The three-way interaction between water volume, watering heterogeneity and belowground herbivory, significantly affected mean plant biomass (Figure 2, Table 5A). The herbivory effects on plant biomass differed between the combinations of water-supply volume and heterogeneity: belowground herbivory interacted with heterogeneous conditions and a small water volume (Figure 2A; Table 5B). When a small water volume was supplied, the plant biomass was larger in the control treatment than in the free treatment under homogeneous conditions and smaller than in the free treatment under heterogeneous conditions (Figure 2A). Under both homogeneous and heterogeneous conditions, plant biomass in the top treatment was the smallest between the three zone treatments. Plant biomass in the free treatment was nearly the same as that in the top treatment with a homogeneous water supply and as large as that in the middle or bottom treatment with a heterogeneous water supply.

**Table 5:** Effects of water volume (V), water heterogeneity (H), and belowground herbivore (B) on plant biomass.

(A) Three-way analysis			
Source	*χ* ^2^	df	P
A	1858.5	1	<0.001
H	23.367	1	<0.001
B	512.59	4	<0.001
A × H	1.084	1	0.298
A × B	32.951	4	<0.001
H × B	31.641	4	<0.001
A × H × B	27.610	4	<0.001
(B) Two-way analysis at small water volume			
Source	*χ* ^2^	df	P
H	4.443	1	0.035
B	28.933	4	<0.001
H × B	32.501	4	<0.001
(C) Two-way analysis at large water volume			
Source	*χ* ^2^	df	P
H	2.096	1	0.148
B	20.968	4	<0.001
H × B	1.304	4	0.861

Under water supply of large volume, plant biomass significantly differed depending on the vertical distribution of a belowground herbivore (Figure 2B; Table 5C). In the treatment with herbivore in the top zone, plant biomass was remarkably small under both water heterogeneity conditions. Plant biomass in the free treatment was the smallest in all treatments with a homogeneous water supply, and plant biomass in the free treatment was slightly larger than in the top treatment with a heterogeneous water supply.

### Mortality of belowground herbivores and its effect on plant biomass

The interaction between water volume and water supply heterogeneity significantly affected mortality of belowground herbivores (*χ* ^2^  = 5.249, df  = 1, *P* = 0.022). With a large water volume, 15 belowground herbivores in the homogeneous treatment died, as did 9 in the heterogeneous treatment (Table 1). With a small water volume, 5 died in the homogeneous treatment and 11 in the heterogeneous one (Table 1). Mortality of belowground herbivores did not differ between the feeding zones (*χ* ^2^  = 0.895, df  = 3, *P* = 0.827).

Mortality of the belowground herbivore significantly affected plant biomass with a large water volume (GLM, *F* = 6.793, df  = 1, *P* = 0.012) but not with a small water volume (GLM, *F* = 0.321, df  = 1, *P* = 0.573). Plant biomass in the pots with herbivore mortality was larger than those without mortality.

## Discussion

Plant mortality occurred only in the treatments in which the herbivore was shallow in the soil to sever the aerial shoot from its root system. This plant mortality caused by belowground herbivory is consistent with our previous study [33]. With heterogeneous supply of small volume water, no plant was grazed at the root base and any plants did not die, probably because low moisture in the surface soil was not suitable for grubs to graze there.

The interaction between available volume and heterogeneity of water supply would change the vertical distribution of the grub in the free treatment. With a small volume of water under a heterogeneous supply, the mean plant biomass in the free treatment that was almost equivalent to those in the bottom and middle treatments, suggest the grubs occurred in the bottom and middle zones due to the low soil moisture levels and large moisture variability. Consequently, the herbivory effects on plant growth varied depending on the availability of water supply.

The vertical distribution of the belowground herbivore was consistent with the previous studies in which belowground herbivores were distributed deep in the soil under dry conditions [18]–[20]. The belowground herbivores may graze fine roots deep in the soil, which may promotes root turnover and enhances the absorption of resources [38]. Belowground herbivory can thus affect plant growth in either a negative or a positive way, depending on the heterogeneity of the water supply. In contrary, with the large amount of available water regardless of supply patterns, belowground herbivory occurred in shallow soil in the free treatment due to enough soil moisture. Therefore, plant biomass was significantly lower than that in the control treatment.

Mortality of belowground herbivores varied depending on available water, which altered plant growth. When the grubs died with enough volume of available water, plant biomass was large because of the negligible loss of roots. When the available water volume was small, herbivore mortality was higher under a heterogeneous water supply than a homogeneous one, which is consistent with previous studies [23]–[26]. However, the effects of herbivore mortality on plant biomass were not significant under a small water volume in this experiment because only few herbivores died.

To our knowledge, this is the first experiment to evaluate the simultaneous effects of available water volume, heterogeneity of water supply, and belowground herbivory on plant growth. The results are consistent with our hypothesis: the availability and heterogeneity of water supply changed the vertical distribution and mortality of belowground herbivores, and consequently plant growth. Therefore, the heterogeneity of soil water supply should be considered in root herbivory studies [15].

Severe climatic events attributable to climate change are already having serious consequences for plants and their herbivores [9], [39]–[42]. We observed high mortality and changes in the vertical distribution of belowground herbivores under the most extreme conditions of water supply (i.e. small volume and heterogeneous supply); these consequently affected plant growth. Therefore, changes in the effects of belowground herbivores on plant growth are likely to occur under the severe weather conditions resulting from climate change.
